# Quantitative assessment of the expanding complementarity between public and commercial databases of bioactive compounds

**DOI:** 10.1186/1758-2946-1-10

**Published:** 2009-07-06

**Authors:** Christopher Southan, Péter Várkonyi, Sorel Muresan

**Affiliations:** 1 ChrisDS Consulting, S-42166, Göteborg, Sweden; 2 EMBL-European Bioinformatics Institute, Wellcome Trust Genome Campus, Hinxton, Cambridge, CB10 1SD, UK; 3 DECS Global Compound Sciences, Computational Chemistry, AstraZeneca R&D Mölndal, S-43183 Mölndal, Sweden

## Abstract

**Background:**

Since 2004 public cheminformatic databases and their collective functionality for exploring relationships between compounds, protein sequences, literature and assay data have advanced dramatically. In parallel, commercial sources that extract and curate such relationships from journals and patents have also been expanding. This work updates a previous comparative study of databases chosen because of their bioactive content, availability of downloads and facility to select informative subsets.

**Results:**

Where they could be calculated, extracted compounds-per-journal article were in the range of 12 to 19 but compound-per-protein counts increased with document numbers. Chemical structure filtration to facilitate standardised comparisons typically reduced source counts by between 5% and 30%. The pair-wise overlaps between 23 databases and subsets were determined, as well as changes between 2006 and 2008. While all compound sets have increased, PubChem has doubled to 14.2 million. The 2008 comparison matrix shows not only overlap but also unique content across all sources. Many of the detailed differences could be attributed to individual strategies for data selection and extraction. While there was a big increase in patent-derived structures entering PubChem since 2006, GVKBIO contains over 0.8 million unique structures from this source. Venn diagrams showed extensive overlap between compounds extracted by independent expert curation from journals by GVKBIO, WOMBAT (both commercial) and BindingDB (public) but each included unique content. In contrast, the approved drug collections from GVKBIO, MDDR (commercial) and DrugBank (public) showed surprisingly low overlap. Aggregating all commercial sources established that while 1 million compounds overlapped with PubChem 1.2 million did not.

**Conclusion:**

On the basis of chemical structure content *per se *public sources have covered an increasing proportion of commercial databases over the last two years. However, commercial products included in this study provide links between compounds and information from patents and journals at a larger scale than current public efforts. They also continue to capture a significant proportion of unique content. Our results thus demonstrate not only an encouraging overall expansion of data-supported bioactive chemical space but also that both commercial and public sources are complementary for its exploration.

## Background

Since the arrival of ChEBI and PubChem in 2004 the development of public domain web-based chemistry databases can justifiably be termed a "big bang" [1-3]. Within the space of five years, the Chemical Structure Lookup Service now claims 46 million unique structures, followed by ChemSpider [4] and PubChem [5], each with collections of 20 million compounds, eMolecules [6] with 10 million and ZINC [7] with 8.5 million. While these are major enabling resources for those working at the interface between chemistry and biology, their content is predominantly aggregated from commercial chemical suppliers. Because only a minority of these compounds can be linked to bioactivity data this can be termed the vendor dilution effect. PubChem is an exception in that it is not only an open chemical information repository but also has a crucial focus on linking compounds to the many types of biological information within the National Center for Biotechnology Information (NCBI), including an increasing amount of public assay data.

The problem of vendor dilution is addressed by specialised databases, both public and commercial, that focus on smaller compound sets that have direct links to documented bioactivity i.e. that specific effects of these compounds in biological systems, ranging from biochemical assays to whole organism studies, have been measured or recorded. For *in vitro *data they can thus include specific links between compounds and the proteins whose activities they modulate. These can be classified as compound-to-assay-to-protein relationships if they are explicitly supported by data within documents. Typically, a journal paper or patent document "D" describes a biochemical assay "A" with a quantitative result "R", for example, an IC50 for compound "C" that define it as an inhibitor of protein "P". The relationship between these five entities of document, assay description, assay result, compound structure and protein identifier (D-A-R-C-P) can be manually extracted by expert curators and organised in a relational format, thereby converting unstructured to structured information. The resultant databases are typically referred to as chemogenomic or large-scale structure-activity relationship (SAR) databases.

Three databases in this study, GVKBIO, WOMBAT (commercial) and BindingDB (public), include curated links of this general type; although there are differences in exactly how each of them is structured and populated. PubChem also contains relationships of this type but these are generally curated by third parties. One example is the depositions from *Nature Chemical Biology *whereby the combined efforts of authors and publishers provide (for subscribers) reciprocal links to compounds where their activity data is specified in the paper. PubChem BioAssays also contains relationships of the A-R-C-P type where assays and their results (A-R) are web-linked rather than document-linked. Interesting exceptions are the 20 assays in PubChem submitted from BindingDB where the data have been extracted from publications.

Databases compared in this work incorporate other types of bioactivity relationships that cannot be detailed here but four important examples should be mentioned. The first is that BindingDB, as inferred from the name, includes directly measured or transformed affinity data i.e. where C-P becomes a "binds-to" relationship. The second example is compounds extracted from the Protein Data Bank (PDB) i.e. where C-P becomes an "is-a-ligand-of" relationship. These are not only captured in PubChem but also selectively in BindingDB and DrugBank for drugs and drug-like compounds. The third example is what could be called bibliographic or co-citation mapping. This can be represented as D-R-C where the document, D, contains information describing some qualitative *in vivo *activity of the compound, e.g. from an animal study or a clinical trial, but no quantitative parameters (A-R) can be extracted. Examples would include the compound names mapped to a drug profile in DrugBank and the compound name-to-MeSH links in PubChem. The fourth is where mapping can link the compound structure to quantitative biological effects *in vivo*, e.g. pharmacological effects, metabolic profiling, or therapeutic efficacy. In cases where the mechanism of action is unknown, these can become compound-centric relationships (D-A-R-C) if the data do not support a direct protein link. The power of data mining across different types of relationships that intersect at the same or similar chemical structures is clear. For example, it can support causality for the biological effects of a particular compound where the SAR and therapeutic mechanism of action in a clinical setting, as well as its efficacy in an animal disease model, can be attributed to specific inhibition of the enzyme target *in vitro*, a binding constant, a PDB structure and linked to its conversion to defined metabolites.

Data extracted into commercial databases in this study are predominantly generated in a pharmaceutical R&D setting. While those databases that map approved drugs to their primary targets can justifiably be designated target-centric it is necessary to be circumspect about such a classification because the protein mappings may infer other mechanistic relationships. The first of these would be non-target compound interactions not directly involved in the therapeutic mechanism of action, such as albumin binding or metabolic turnover by P450s. The second could be termed anti-target effects, such as hERG binding. The third type, commonly referred to as off-target interactions (or polypharmacology if this is a designed property), are derived from compound specificities recorded from cross-screening results. An example used in this work is the extraction of published protease inhibitors for BACE1. While these documents are directed towards the discovery of therapeutic candidates for Alzheimer's disease, they often include inhibition data against the paralogue BACE2 even thought this is not currently considered a drug target.

There are number of recent publications describing the proliferation of public and commercial cheminformatic resources [8-10]. However, the quantitative comparison of content has so far been limited to two recent studies suggesting both sectors were offering unique content and functionality for bioactive chemistry [1,11]. This work describes an update on the first of these two studies that shows significant changes in two years. We have added new sources, collated information on compound-to-protein mappings and calculated compounds-per-document.

## Database descriptions and processing

The criteria by which we selected databases for inclusion in this study are outlined below:

1) They include curated compound-to-activity-to-protein mappings and/or some other focus on bioactivity data.

2) They have been continually updated.

3) They have an established utility for drug discovery within AstraZeneca.

4) We were familiar with their data structures, content and sources.

5) For public databases we were able to download their entire compound content, either directly or via PubChem.

6) For commercial databases subscribed to by AstraZeneca we had complete internal access to their compound content (online-only sources were therefore excluded).

7) Their formats allowed file conversions for structure filtration.

8) The public databases had reciprocal links to PubChem and could therefore be selected as a submitting source within Entrez.

9) None of the commercial products included any proprietary information i.e. all structures and associated data were extracted from published documents.

This study includes some changes from the 19 databases and subsets used previously [1]. Two commercial databases were dropped because they had not been updated internally. One public drug set was also omitted because it had not been updated. Three new databases were added, a new Clinical Candidate product from GVKBIO (commercial) as well as ChEBI and BindingDB (public) because they had significantly increased their content and functionality since our previous study. The BioAssay actives and PDB ligand subsets were changed as described below.

This section includes a brief summary of the databases and subsets. We have included self-reported statistics of their content from September 2008 or by selecting them as a source in PubChem to obtain a compound count. More detailed information is available from the source references, database help facilities, and documentation provided on the company websites.

### PubChem

PubChem [5] is the NCBI public informatics backbone for the NIH Molecular Libraries Initiative focused on small molecules as systems biology probes and potential therapeutic agents. It consists of PubChem Compound (unique structures), PubChem BioAssay (assay results), and PubChem Substance (samples supplied by depositors). Because the identifiers are discussed in Results it is necessary to distinguish between compounds and substances. A PubChem Substance Identifier (SID) represents distinct chemical structures from each submitting source. The PubChem Compound Identifier (CID) is essentially a merge of those SIDs with an identical structure, i.e. a reduced-redundancy representation. The statistics are 19.3 million compounds with 44.5 million links from 65 submitting sources i.e. on average each CID is a merge of 2.3 SIDs. Of the compounds, 784,039 have been tested in 1,190 assays. This connectivity is integrated into the Entrez bioinformatic databases. The website includes comprehensive descriptions of data structures, content and mining tools.

While the compound structures *per se *are all public it should be noted that PubChem includes structures from commercial databases but access to the out-linked information requires a subscription. The largest submissions of these are the 5.7 million substances from DiscoveryGate [12] and 2.7 million from Thompson Pharma [13]. The former is essentially a cross-matching process, i.e. out-links are only added when they match pre-existing submissions from another source, whereas the latter includes *de-novo *deposition. Thus, the Thompson Pharma component represents a "commercial-within-public" data source. While this is of undoubted value for subscribers it does have a confounding effect on our extrinsic comparisons with commercial databases because it was deposited between the 2006 and 2008 snapshots. Therefore, some of the increased overlap between PubChem and commercial databases, recorded in the Results for 208, can be ascribed to this. It should be pointed out that, while for non-subscribers the value of PubChem structures without informative out-links is limited, the 2.2 million entries that have both DiscoveryGate and Thompson Pharma SIDs do have some utility because of their implied bioactivity.

### DrugBank

The 2008 release of this public database includes, as specified on their website, 4,765 compounds divided into 71 nutraceuticals, 128 biotech (protein) drugs and 4,637 small molecule drugs. Of these, 1,485 are approved drugs, 3,243, experimental drugs, 188 illegal drugs and 64 withdrawn drugs. Each DrugCard entry contains over 80 searchable data fields, with half being devoted to drug and chemical data, and the other half to target-centred information [14]. The SD files were downloaded directly from the website as it is not yet possible to separately select the approved or experimental sets from within PubChem.

### BindingDB

This public database currently contains experimentally determined enzyme kinetic data, measured or derived binding affinities of protein-ligand complexes, and protein targets for small molecule ligands [15]. These data are manually extracted from journals by curators but some sets have been submitted by authors. This database has a focus on proteins that are drug targets, candidate drug targets and/or in the Protein Data Bank. Identifiers for curation or submission sets, predominantly as PubMed IDs from publications, are given on the website along with September 2008 figures for 23,931 compounds and 45,363 Ki or IC50 data values. When selected from PubChem, 23,021 CIDs were retrieved.

### ChEBI

Chemical Entities of Biological Interest (ChEBI) is a public database of endogenous and synthetic bioactive compounds [16]. It also includes a chemical ontology classification, whereby the relationships between molecular entities or classes of entities and their parents and/or children are specified. It incorporates compounds specified in the Integrated relational Enzyme database (IntEnz) of the European Bioinformatics Institute that contains the recommended Nomenclature and Classification of Enzyme-Catalysed Reactions [17]. It also incorporates KEGG COMPOUND that is part of the LIGAND composite database of the Kyoto Encyclopaedia of Genes and Genomes (KEGG) [18]. Release 48 of ChEBI specified 15,548 annotated entities on their website and the PubChem selection provided 10,192 CIDs. While ChEBI does not currently have direct bioactivity links to journal articles, they have recently introduced automatically generated cross-reference where compound names are matched with those extracted from patent documents via the European Patent Office.

### WOMBAT

WOrld of Molecular BioAcTivity (WOMBAT) is a commercial database that includes specific links between compounds and sequence identifiers for the proteins against which these compounds have been shown to be active [19]. In addition to the compound structures and activity values, the species in which the tests were performed, an activity classification of the compound as well as additional calculated properties are included. From the website, WOMBAT 2008.1 contains 220,733 entries (192,529 unique SMILES) captured from papers published in medicinal chemistry journals between 1975 and 2007. Approximately 88% of the targets named in source documents have been assigned unambiguously to SwissProt IDs.

### GVKBIO

This study includes four separate commercial products from GVKBIO, two of which have been merged into one internal database in AstraZeneca [20]. This merge was between the MedChem database of compounds extracted from medicinal chemistry journals and the Target Inhibitor Databases that are divided into nine sections, GPCRs, proteases, kinases, ion-channels, NHRs, phosphatases, transporters, phosphodiesterases and lipases. Compounds in these nine target databases have been extracted from both journals and patents. Compounds from the latter are selected from all exemplified claims with specified activity data but capped at 500 for ranged activity values or 200 examples for qualitative results. This merged database is referred to as GVKBIO in the results and has been described in outline [21]. The current content statistics from direct queries are 3,344,068 compound links, 2,153,846 unique structures and 10,314,951 activity values extracted from patents and papers. The GVKBIO on-line portal, GOSTAR, contains an equivalent merge and includes the two products below plus additional sources.

The GVKBIO Drug Database (GVKBIO DD) contains data on drugs approved by the US Food and Drug Administration (FDA) and other authorities. This data has been extracted from pharmacological journals and other sources. It includes protein target information, therapeutic classification and pharmacokinetic parameters. The Clinical Candidate Database (GVKBIO CCD) includes compounds which are in various stages of drug development with associated biological, pharmacological, target identity and clinical pharmacokinetic information extracted from journals covering clinical development of new drugs and patents. It includes FDA-approved compounds, failed or discontinued clinical candidates, discontinued drugs, late-stage research compounds, those reported in phase I, II and III trials as well as available metabolite information.

### MDDR

The MDL Drug Data Report (MDDR) is a commercial database by Symyx/MDL that covers published documents, meeting reports and congress proceedings [22]. It contains both the structures of compounds and their derivatives. Compound-linked information includes bioactivity, therapeutic classification, detailed patent information, literature references, company names, compound codes, generic names, trade names and development phase. The 2008.1 release contained 185,844 compounds.

### DNP

The Dictionary of Natural Products (DNP) from Chapman & Hall/CRC is a commercial database of natural products [23]. It arose as a daughter product of the Dictionary of Organic Compounds (DOC) that has been in book form for many years. It includes data on chemical, physical and biological properties of compounds; systematic names, common names, CAS registry numbers, literature references; structure diagrams and connection tables.

### Database Subsets

In addition to using complete compound collections we selected subsets to provide insights into more specific areas of bioactive coverage. The PubChem subsets were extracted as PubChem CIDs and SMILES strings by using the Entrez query and download facility. They were subsequently cross-matched to the entire set downloaded from PubChem.

**1. PubChem Prous**: The Prous *Drugs of the Future *Journal publishes monographs with information on new drug compounds in development. These were retrieved by searching PubChem Compound with the Entrez limit: "Prous Science Drugs of the Future" to give 4,622 CIDs. While the out-link between the compounds and the documents are explicit in PubChem, this is not yet reciprocal i.e. there are neither links from articles out to PubChem CIDs nor between compounds and sequence identifiers of the targets that are, in most cases, identifiable in the context of the document.

**2. PubChem PDB**: While in our previous study we had retrieved a specific set of small molecule ligands using the data source "SMID", the update status of this set was unclear. We therefore chose a larger set (PubChem compound, Limits: Protein 3D Structure) that gave 12,756 records. This is an upper limit that includes many entries that would not be classified as bound ligands. By using the rule-of-five [24] chemical property filters in PubChem this was reduced to a set of 5,706 compounds that should be predominantly drug-like ligands.

**3. PubChem actives**: The PubChem BioAssay database includes purified protein activity assays, cell-based screens and other types of measurements. The query used in our previous study, "active in any bioassay", was used to retrieve 11% of all compounds screened at that time. However, it was subsequently brought to our attention that this included not only likely false positives from primary screens but also compounds testing positive in various molecular property assays such as fluorescence. This "active in any bioassay" subset has now increased to over 20% of all tested compounds. Because there are no options to de-select molecular property assays the following filters were used to enrich for authentic bioactives within the limitations imposed by standard Entrez queries. The first was to select only those bioassays that had a protein target, the second was those with specified testing concentrations (typically for IC50s) and the third was to restrict to confirmatory rather than primary screens. This produced 195 assays from 71 unique gene products. Retrieving actives from these assays gave just over 21,000 compounds (this actually selects those active in any assay but it was not possible to select only from the 195 specific assays). The final filter was to select compounds with an active concentration of 1.0 μM or below. This combination produced 4,300 compounds that should be predominantly the most potent actives from confirmatory dose-response biochemical assays against purified protein targets.

**4. PubChem pharmacology**: The MeSH index of PubMed includes the category "pharmacology" for drugs and other exogenously administered bioactive compounds. It includes activators and inhibitors of physiological or biochemical processes and other pharmacological mechanisms of action. As MeSH-defined compounds are linked into PubChem, the Entrez query "pharmacological action" was used the retrieve these. Surprisingly this compound subset had decreased since our previous study from 12,038 to 10,785. It turns out that this category had undergone a clean-up whereby spurious mappings caused by naming errors in some submissions had been corrected.

**5. PubChem MLSMR**: The new PubChem subset we included was the NIH Molecular Libraries Small Molecule Repository (MLSMR) collection. This is used for screening by the NIH Molecular Libraries Screening Centre Network (MLSCN). The Entrez query in PubChem Compounds for this set retrieved 300,585, although screening results have so far been deposited for only 232,839 of these. While some individual assays use smaller subsets rather than the entire collection, these compounds have been used in 727 MLSCN assays. The results generated contain increasing amounts of cross-screening data as the assay collection expands. In this case 80% of the compounds complied with the rule-of-five filters [24]. This MLSMR subset is therefore the conceptual equivalent of a pharmaceutical company screening collection.

**6. GVKBIO journal**: This subset of GVKBIO compounds from the MedChem and Target databases was selected by field code "journal" in the database.

**7. GVKBIO patent**: Subset of GVKBIO compounds from the Target databases selected by field code "patent" in the database.

**8. BACE1**: From the GVKBIO database we extracted a single example set of compounds that have been tested for their activity against one specific molecular target. We chose BACE1 for three reasons: 1) it has a clearly delineated research phase as a validated drug target for Alzheimer's disease [25], starting from 1999, 2) it has a paralogous gene family of only two members, BACE1 and BACE2, and 3) with over 5,000 compounds retrieved using the Entrez Gene ID 23621 for human BACE1, it was close to the median compound-to-human target numbers in the GVKBIO database that are distributed between 1 and over 50,000. By inspection of the documents linked to these compound records it was established that, as expected, these compounds had been tested in biochemical assays for BACE1 substrate turnover and were predominantly selective inhibitors of the protease although some with low activity levels were included in the screening result sets.

**9. DrugBank approved**: Approved small molecule drugs were downloaded directly from the site as set of SD files.

**10. DrugBank experimental**: Experimental drugs including unapproved, withdrawn, illicit drugs, enzyme inhibitors and potential toxins. These were downloaded directly from the site as set of SD files.

**11. MDDR launched**: A subset from MDDR selected by field code "launched" in the SD files.

### Database Processing

For both the extraction of subsets from PubChem that required multiple steps (e.g. active compounds and PDB ligands) and the generation of complimentary data by various combinations, the Entrez facility was used. Queries were done at the compound level with particular sources or subsets selected via the "Limits" tab. Following the sequential selection of multiple compound sets the appropriate Boolean combinations were selected via the "History" tab and performing aggregations using OR as well as determining overlaps with AND.

All commercial compounds were downloaded as SD file format from their internal Oracle databases within AstraZeneca. DrugBank was downloaded from its website as SD files. All SD files were converted into SMILES strings. Where indicated above, other public collections or subsets were selected via the appropriate Entrez limit in PubChem and downloaded directly as SMILES strings. After conversion of all sources to SMILES, stereochemical information was removed in order to avoid differences between representational conventions and thereby allow a direct all-against-all database comparison [26]. The non-isomeric SMILES were further processed to determine the number of unique structures in each database by the following four-step filtration procedure:

1. Normalisation of each structure by removing small fragments, such as counter ions, water, and neutralising remaining charges.

2. Derivation of a canonical tautomer using LEATHERFACE, an in-house molecular editor based on SMARTS rules [27].

3. Generation of unique molecular hashcodes [28].

4. The retaining of unique structures by comparing the molecular hashcodes.

The same molecular hashcodes were subsequently used to identify the overlap between databases. All in-house scripts and software used for this study are based on the OpenEye toolkit [29].

### Compound, Document and Protein Counts

The filtration process described above produced final chemical structure counts for all sources and subsets. These numbers are in Table 1 of the results section. Document counts are the total number of discrete publications from which the compound structures and protein identifiers were extracted. For the GVKBIO products these were generated from in-house queries restricted to patent numbers and PubMed IDs. In other cases document counts were obtained from the web sources or suppliers information. While we expect these predominantly to be in PubMed the numbers for BindingDB and DNP are not explicitly PubMed ID counts. Protein counts for the GVKBIO products were also generated by in-house queries of distinct EntrezGene IDs with or without human in the annotation field as an additional restriction. For WOMBAT the protein counts were supplied to us. In the case of BindingDB and DrugBank we used the UniProtKB/Swiss-Prot database cross-reference for the protein counts rather than figures from the websites as this enabled the additional restriction to human proteins. For the assay targets linked to the PubChem actives and the PubChem PDB compound set, the RefSeq identifiers were obtained but species selects for these were not available via a standard PubChem query.

**Table 1:** Database update numbers.

Dataset	Oct 2006	Oct 2008	2008–2006	2008–2006 [%]	Filtration 2008
**GVKBIO**	1488288	2054151	565863	38%	-8%

**GVKBIO Journals**	542858	658198	115340	21%	-8%

**GVKBIO patents**	1034548	1484218	449670	43%	-7%

**GVKBIO DD**	1933	3675	1742	90%	-4%

**GVKBIO CCD**	n/a	8864	n/a	n/a	-1%

**GVKBIO BACE1**	n/a	5228	n/a	n/a	-11%

**GVKBIO BACE1 journals**	n/a	389	n/a	n/a	-6%

**GVKBIO BACE1 patents**	n/a	4901	n/a	n/a	-11%

**WOMBAT**	128120	180856	52736	41%	-18%

**PubChem**	7268193	14965539	7697346	106%	-23%

**PubChem Prous**	3318	4652	1334	40%	-2%

**PubChem PDB**	5626	5706	n/a	n/a	-8%

**PubChem actives**	35671	7472	n/a	n/a	-3%

**PubChem pharmacol**	6070	5311	n/a	n/a	-63%

**PubChem MLSMR**	n/a	233284	n/a	n/a	-1%

**PubChem BindingDB**	n/a	24203	n/a	n/a	-4%

**PubChem ChEBI**	n/a	7428	n/a	n/a	-31%

**DrugBank**	3723	4545	822	22%	-7%

**DrugBank approved**	1018	1341	323	32%	-3%

**DrugBank experimental**	1737	2999	1262	73%	-6%

**DNP**	131831	144383	12552	10%	-26%

**MDDR**	159867	176600	16733	10%	-4%

**MDDR launched**	1118	1435	317	28%	-5%

This shows reductions in the compound figures after filtration in this work (2008). For those data sources that were also included in our previous publication the changes (2006 – 2008) are shown in absolute numbers and as a percentage. The reasons for the decrease in two of these are explained in the methods section. New sources that are not applicable to 2006/2008 changes are labelled n/a.

## Results and discussion

### Post-Filtration number reductions and database increases

The growth rate of the databases and comparable subsets we have used in this and our previous study are shown in Table 1, together with the filtration results from 2008. These latter figures generally show a significant reduction compared to the self-reported compound counts from the sources but it should be borne in mind that these may be defined in different ways. Because of the inherent complexity of chemical structure comparisons, the challenges of standardising representations, and the difficulties of quality control for large compound collections, none of these sources are likely to claim their numbers as a standard of truth. For the same reasons we would not propose our numbers as an absolute normalisation. Nevertheless, this filtration process has proven robust and consistent in our hands for standardising comparisons between compound collections over a number of years. The use of subsets provides internal controls and, in all cases, produced exact number matches i.e. the subsets added up the parent set. As evidence for the precision of these methods, some of the results (e.g. the BACE1 set) show single-digit compound overlaps that have been verified by manual inspection.

Establishing exactly what types of representational heterogeneity have contributed to the filtration reductions in Table 1 is not only outside the scope of this work but also requires a level of process detail that not all vendors are prepared to disclose. Nevertheless, because it is a) numerically the largest source, b) has a level of technical transparency where all internal links can be checked, and c) it exemplifies important aspects of the general case, the results from PubChem can be expanded briefly. As mentioned in Methods, on average, each CID is a merge of 2.3 SIDs. However, while it is crucial to preserve the individual SID representations, as supplied by the individual submitters, heterogeneity in these can feed through to a multiplicity of CIDs. These can include mixtures, salts and instances where different tautomers may, or may not, be specified in different SIDs for the same compound. An example would be the independent submissions of a drug formulation as a mixture, each of the single components and two different salt forms of just the active compound. This would result in, according to the PubChem merging rules, five CIDs that would have been filtered in this work to just two structures.

While these factors have resulted in a 23% overall reduction in PubChem after filtration, it was surprising to note in Table 1 that the Pharmacology subset showed the largest reduction from any source of 63%. It turns out that this subset has a high SID: CID ratio of 13:1. It therefore includes different salts, protonation states and stereoisomers that have been mapped to the same MeSH chemical name. Consequently, this accumulation of SID heterogeneity has spawned many CIDs linked with those MeSH terms which have collapsed during our filtration step to a much smaller number of unique parent structures.

All the independent sources in common between our 2006 and 2008 studies have expanded (the two instances of subset contraction are explained in Methods). The largest increase was in PubChem that effectively doubled in size. The submission of 17.2 million substances from ChemSpider at the end of 2007 that merged into 16.7 million CIDs was clearly a major contribution, although the absolute increase in CIDs from this source was only 6.2 million. The next largest increase at 73% was the DrugBank experimental subset that underwent a release update at the beginning of 2008. The remaining growth rates, in the range of 10% to 30%, are more modest but these particular sources are based on manual document curation, rather than bulk submissions. It is interesting to note that the three sources of approved drugs, DrugBank approved, MDDR launched and the GVKBIO Drug Database, show similar growth rates in the region of 30%. The absolute increases of over 300 seem high since the recent annual FDA approval rates have only been in the 25–30 range, suggesting these sources have processed a backlog over this period [30].

### Compounds-per-protein and compounds-per-document counts

While there are caveats associated with quantifying and comparing these from different databases, Table 2 shows the numbers obtained for selected parameters that could be calculated. It is noticeable that, while the numbers of compounds-per-journal article are comparable, the compounds-per-protein show a trend in order of increasing numbers of documents. This is explicable because the same protein identifier would occur in many documents, particularly for popular targets, and therefore the rate of inclusion of unique protein identifiers would not be directly proportional to the number of documents extracted. Conversely, authors predominantly publish new chemical structures tested against previously established targets so the numbers of these would accumulate as more papers were extracted. For the GVKBIO DD and CCD the ratios are essential reversed i.e. large numbers of documents are linked to a smaller number of compounds and targets This many-to-one relationship is the expected consequence of extracting the numerous publications that constitute the data corpus accrued over many years for established primary targets, launched drugs and late-stage candidates. For MDDR the compounds-per-document (patents and papers in this case) lies between the two extremes.

**Table 2:** Compounds-per-protein and per-document.

Database or subset	Document count	Protein ID type	Total proteins	Human proteins	Cpds-per-protein	Cpds-per-document
GVKBIO	87747	Entrez Gene	3292	1468	604	22

GVKBIO journals	51810	Entrez Gene	2660	1146	239	12

GVKBIO patents	35937	Entrez Gene	1765	952	815	40

GVKBIO DD	26825	Entrez Gene	733	339	5	0.14

GVKBIO CCD	27286	Entrez Gene	1224	610	7	0.32

WOMBAT	10205	Swiss-Prot	1979	1095	91	18

DrugBank	n/a	Swiss-Prot	1625	1356	3	n/a

PubChem actives	n/a	RefSeq	72	n/a	104	n/a

PubChem PDB	n/a	RefSeq	818	n/a	14	n/a

BindingDB	1142	Swiss-Prot	297	97	112	19

MDDR	137754	n/a	n/a	n/a	n/a	1.4

DNP	7765	n/a	n/a	n/a	n/a	18

Column three is the type of protein identifier used for the count of all species (column four) and human proteins (column five). In columns six and seven the filtered compound totals are taken from Additional file 1. The compound ratios are calculated with respect to total proteins and documents. For boxes labelled n/a the information was either not applicable or not available. For reference we have included a compounds-per-protein calculation for the PubChem actives subset even though there are no document-protein links analogous to the other sources.

The noticeable differences between patent and journal extractions in Table 2 are that the later produce fewer compounds but more sequence identifiers. The fact the exemplified compounds in patents outnumber those in journal papers is unsurprising and this would be even higher if it were not for the fact that GVKBIO cap the examples extracted from patents with qualitative biological data. The reason patents produce less human sequence identifiers is that smaller target classes, outside the "big-nine" listed in Methods, are not prioritised for extraction. The reasons for less total sequence identifiers in patents are not only the target class restriction but also because activity data against non-human proteins is more commonly included in journal articles than patents.

Because DrugBank and BindingDB both have cross-references in UniProtKB/Swiss-Prot the Sequence Retrieval Service (SRS) can be used to ascertain proteins common to both databases [31]. This gives the result of 39, of which 35 are human. However, comparing these two databases also illustrates some of the caveats. BindingDB declare their total sequences mapped to compounds at 515. While there may be additional complications related to accession numbers one of the reasons for an apparent under-count when mapping to Swiss-Prot IDs is the capture of data for the many single amino acid protein variants used in binding studies that have the same Swiss-Prot ID. Conversely, the number of primary targets for the compounds in DrugBank is a significant over-count for reasons explained in the publication [14]. In the context of protein identifiers within databases the important utility of being able to perform BLAST searches against the actual sequences (and link back to compound data) is currently limited to DrugBank, BindindDB and the protein targets in PubChem BioAssays.

The results in Table 2 can be viewed in the context of established and predicted druggable genes. The lower limit can be defined as the 207 human primary protein targets of approved small-molecule drugs and 41 for pathogens [32]. The upper limit, extrapolated from this drugged set, has recently been estimated at 2,200 human genes [33]. Other key numbers include the 836 human targets with some kind of documented chemical modulation starting point [34]. Last but not least, was the prescient estimate that the human genome could include 600–1,500 tractable and therapeutically effective targets [35]. It is of interest to note that numbers from the GVKBIO Med Chem and Target databases of 1,468 human protein-compound links are already approaching this upper limit although, as mentioned in the introduction, this number includes a proportion of non-targets. The GVKBIO DD human protein count slightly exceeds known primary drug targets because they also include some protein identifiers from cross-screening data. The higher protein count in GVKBIO CCD can be attributed to two causes. The first is the inclusion of proteins that are not yet targeted by approved drugs, i.e. new targets, and the second is also the inclusion of cross-screening data.

The absence of figures in Table 2 for some databases in the study needs clarification. ChEBI does include protein names mapped to compounds in their curated ontology (e.g. hydroxymethylglutaryl-CoA reductase inhibitor) but these are currently limited in number. It also includes automatically generated cross-references to a number of external databases. This includes UniProtKB entries for all protein names associated with that particular compound but these are not curated links. Protein names from the literature and inferred metabolic interactions may be associated with structures in DNP but there is no formal mapping of these relationships. Compounds in MDDR have links to therapeutic or generic activities. While it is possible to extrinsically cross-map many of these to specific protein identifiers, these are not curated links in the current data structure [36].

### The Comparison matrix

We should point out that some of our explanations outlined below for the patterns of overlaps and their shifts from our previous publications are speculative. There are two reasons for this. The first is that, within the constraints of this work, it has only been possible to make a limited number of additional comparisons and manual inspection of selected entries to try to establish the basis for differences. The second reason is that, compared to the many journal articles and presentation slide sets describing public databases, detailed information as to how commercial databases are populated is not always available. Notwithstanding, we have acquired extensive knowledge about their content, including feed-back from vendors. This informs our judgment despite the paucity of citable documentation. We thus consider our inferences not only to be an informative part of this work but also to be testable by further analysis. The results, presented as Table Three in Additional file 1, will be referred to as column numbers but these intersect with the same row numbers in the matrix.

Column 1 shows the GVKBIO database has increased since 2006 to just over 2 million compounds and is the commercial source with the highest unique content in this study. For the subsets in columns 2 and 3 the patent: journal ratio is 2.28:1 with just under 6% of patent compounds overlapping with those appearing in journals, presumably subsequent to the patent publication, although some could be non-proprietary bench-marking compounds. To our knowledge, such an important measurement has not been reported elsewhere, except in our previous study where it was 10%. As to whether this decrease really represents acceleration in patent chemical structure output relative to journals is perhaps too early to establish. In fact, the overall numbers of patent-specified structures eventually appearing in journals is likely to be higher than these figures for three reasons. The first two have been already mentioned in the GVKBIO database descriptions, namely the necessity to cap compound structure extractions from large applications and limit the target classes covered by selected patent documents. The third reason is that not all journals that might include some compound bioactivity data from pharmaceutical R&D are extracted.

In column 4 it can be see that, only 38% of the drug compounds from GVKBIO DD are represented in patents but 75% overlap with GVKBIO journal compounds. The same trends can be seen not only for the other drug collections (columns 16 and 20) but also for the clinical candidate compounds from GVKBIO CCD (column 6) i.e. that only 34% of the compounds are in patents but 62% overlap with those from papers. Similar ratios can also be detected in the Prous *Drugs of the Future *subset as an independent development compound source i.e. with a 75% overlap with GVKBIO papers but 37% for patents. While it would seem unlikely for clinical candidates or approved drugs not to have their structures claimed in patents the explanations we propose for this have already been given above, namely the extraction cap and target class restriction for patents. The 89% overlap with GVKBIO CCD establishes that the majority of clinical candidates are being deposited into PubChem, possibly via other commercial sources such as Thompson Pharma, but further subset comparisons would be necessary to establish this.

Column 6 establishes that GVKBIO Journals covers 94% of WOMBAT, just one percent more that in our 2006 study, showing the concordance between these independently curated sources remains high, even though both have expanded significantly (Table 1). Column 7 shows that PubChem covers the largest proportion of the commercial databases in this study, with the exception of WOMBAT, where the 75% overlap with PubChem is lower than the 94% with GVKBIO. From the comparison between GVKBIO and PubChem the coverage of the former by the latter has increased from 29% to 44%. Most of this increase has come from GVKBIO Patents where PubChem now overlaps with over 0.63 million compounds, almost doubling from 2006. There are two likely sources for this increase. The first is Thompson Pharma that also includes patent-extracted compounds; the second would be via ChemSpider from the SureChem online patent database [37] that automatically converts names to structures from patent documents. However, our results show that the patent-derived coverage by GVKBIO not in PubChem has risen from 0.79 million in 2006 to 0.85 million in 2008.

The PubChem Prous compounds in column 8 have the highest overlap with GVKBIO at 79%, even though *Drugs of the Future *is not currently a source journal. An explanation is that compound-sequence links have been picked up from the primary literature before appearing in the Prous review articles. In this context, the 50% coverage by MDDR seems low. The PDB ligand set in column 9 shows the highest coverage of 50% in GVKBIO followed by 28% in DrugBank. An unexpected result from the PubChem actives in column 10 was that 32% are not in the MLSMR screening collection. While there are certainly submissions to PubChem BioAssay for protein targets run against other screening collections it also turns out that, for some of the confirmatory assays the chemical space around selected primary hits has been expanded by the acquisition or synthesis of new analogues not in the original collection. While they should eventually be added, there is a time lag in this process.

In column 11 GVKBIO has the highest overlap with the PubChem pharmacology subset at 81%. An explanation is that many compounds whose activity *in vitro *is published are also tested *in vivo *and therefore eventually indexed in MeSH. In column 12 the MLSMR collection has little overlap with other sets which, doubtless, reflects the diversity emphasis of the acquisition strategy [38]. However, the overlaps also suggest the collection still has some way to go in regard to the declared objectives of increasing the content of known drugs and natural products because it has only about 50% of the former (columns 4,16 and 20) and less than 0.5% overlap with the latter (column 18). It is also noteworthy that the GVKBIO overlap of just over 2% in the screening collection, rises to 19% in the selected actives (column 12). This suggests an enrichment of compounds with reported bioactivity, despite the fact that the MLSCN pilot phase has tended to screen different protein targets than those represented in the GVKBIO document sources that are predominantly derived from pharmaceutical R&D activities.

The results in column and row 13 include BindingDB for the first time. GVKBIO journals cover 92% of its content. The 8% difference could be in part due to experimental thermodynamic binding data captured in BindingDB that is not one of the assay data types typically extracted by GVKBIO. Column 14 shows the coverage of ChEBI. While GVKBIO shares the highest proportion of content at 44% the low coverage of other databases point to unique content of enzyme intermediates and metabolites. While the 36% overlap shows some of these are in DNP the metabolite coverage is supported by a 56% overlap between ChEBI and KEGG (from a comparison done within PubChem). ChEBI also contains 25% of DrugBank.

Columns 15, 16 and 17 are the DrugBank total, approved and experimental drugs respectively. The total exceeds the number represented within PubChem by 175 because we downloaded these directly from DrugBank. The 68% overlap with GVKBIO is predominantly with journal content rather than patents. Of the approved drugs 86% are in PubChem pharmacology but only 66% in GVKBIO patents. Overlaps between DrugBank and other drug sets are reviewed below. Column 18 shows that the natural product structures have a 56% overlap with PubChem and 12% with GVKBIO. The figures in column 18 suggest (but would need a Venn-type analysis to confirm) that DNP is the commercial source with the second highest unique content in this study. Columns 19 and 20 show the split between MDDR total and launched. The total shows 63% overlap with PubChem and 45% with GVKBIO. The overlaps for MDDR launched are detailed in a later section.

Columns 21 to 23 are the set of BACE1-linked compounds retrieved from GVKBIO. The number from patents is 12.6 times that from journals, confirming the importance of patents as a published source of compounds directed against drug targets. WOMBAT and BindingDB have extracted the same compounds from journals, at 154 and 55 respectively. While these would be expected to be research compounds for BACE1 inhibition there are also matches in GVKBIO DD and GVKBIO CCD (rows 4 and 5). Inspection of these individual compounds revealed two explanations. One of the intersects with GVKBIO CCD was PubChem CID 11537623. This is a BACE1 inhibitor that, as a development candidate, had been captured in GVKBIO CCD. One of the intersects with GVKBIO DD was PubChem CID 5493444, the approved Novartis rennin inhibitor Aliskiren. This is clearly a case of cross-screening against the same target class. This also turns out to be the same compound that is responsible for the single match between the BACE1 set, DrugBank approved and MDDR launched.

### Venn-type results for selected databases and subsets

**Figure 1:**
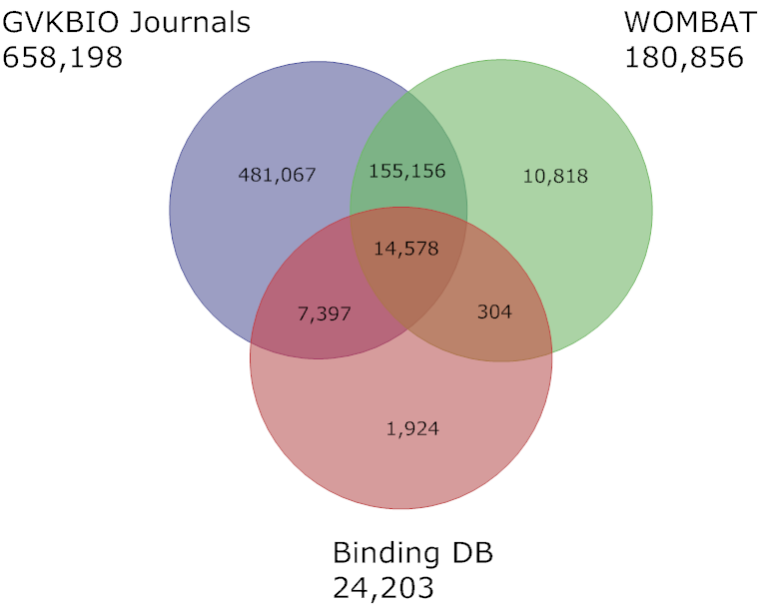
**Compounds extracted from Journals**. A Venn diagram showing the content overlap and differences between the databases or subsets that contain compound structures extracted from medicinal chemistry journals. The data source name and compound totals are given outside each of the three circles.

A limitation of the data in Additional file 1 is that truly unique content needs to be defined in terms of a Venn-type series of B+C+D-A, A+C+D-B and so on. This was performed on three subsets with common themes and in two of these cases we can make a comparison with equivalent data from the previous study. As a consequence of our first inclusion of BindingDB in this publication, the Venn diagram in Figure 1 is able to show overlaps and unique content between three databases that operate a conceptually similar curation model. The concordance of 177,435 between pairs is evidence for the impressive consistency of expert chemical structure extraction from an overlapping document corpus, in this case predominantly medicinal chemistry papers, by three independent curation teams on three different continents, India for GVKBIO, Romania for WOMBAT and USA for BindingDB. Considering the document ratio of approximately 50:9:1 the larger unique set in GVKBIO journals is unsurprising. The non-overlaps in the two smaller databases are likely to be due to differences in selectivity from journal coverage and/or in the choice of compounds extracted from particular articles.

**Figure 2:**
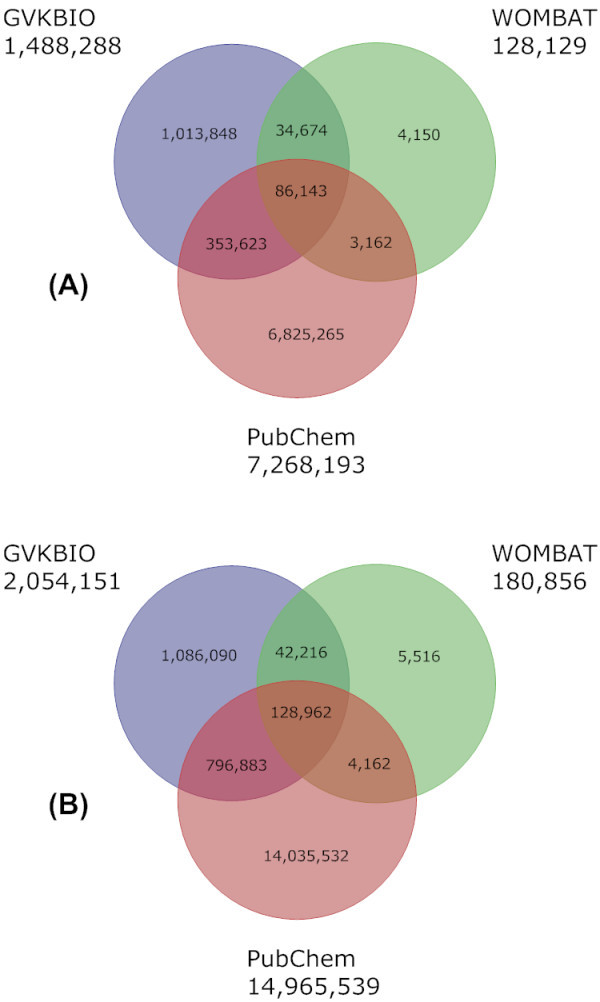
**Comparison of GVKBIO, WOMBAT and PubChem**. A Venn diagram showing the content overlap and differences between GVKBIO, WOMBAT and PubChem. The older 2006 versions are shown in (A) and 2008 from this publication in (B). The data source name and compound totals are given outside each of the three circles.

The diagram in Figure 2 also includes WOMBAT but compares to GVKBIO and PubChem across an approximate 2-year interval over which the three-way overlap has increased. This defines a potentially high-value consensus bioactive subset because these compounds not only show concordance between independent sources but may also link to additional information and/or screening data in the PubChem system. The second trend over the two years is that while the number of compounds unique to GVKBIO is still over a million there has been a doubling in the PubChem overlap due to the increase in patent-derived compounds entering PubChem as shown in Additional file 1.

**Figure 3:**
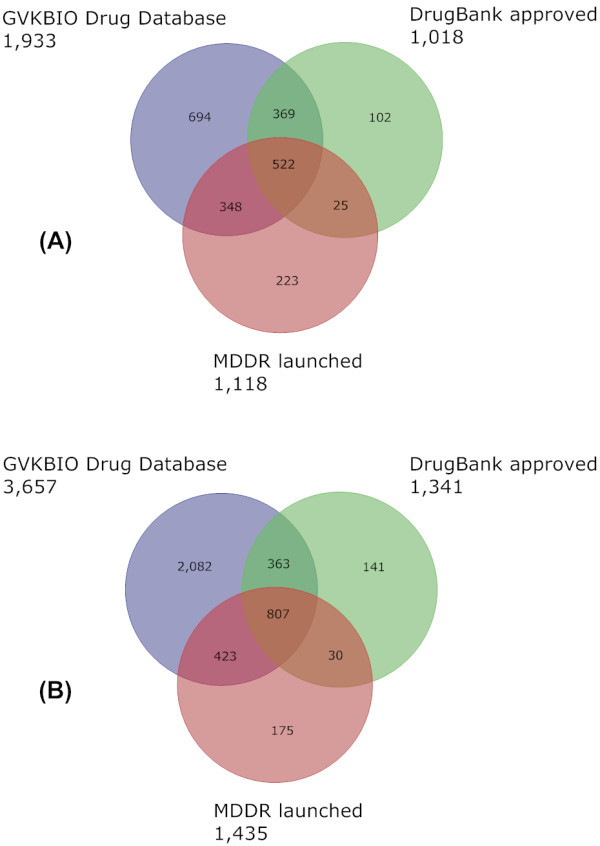
**Comparison of drug databases**. A Venn diagram showing the content overlap and differences between GVKBIO DD, MDDR launched and DrugBank approved. The 2006 versions are shown in (A) and 2008 from this publication in (B). The data source name and compound totals are given outside each of the three circles.

Figure 3 shows the Venn diagram for the database and two subsets that capture approved drugs, MDDR launched, GVKBIO DD (both commercial) and DrugBank approved (public), for this and the previous study. While the concordance between these three sets has increased from 522 to 807 over two years, we would expect this three-way overlap to be approximately 1,300, although the pair-wise overlap is 1,623. One possibility is that extraction from different sources is the cause of the representational differences. Further investigation to confirm this could be important given the lack of an officially authenticated set of standardised compound structures from the FDA and/or other national approval bodies. Numbers have been recently proposed as 1,323 from the FDA Orange Book but without structures [39]. Drugs@FDA also has a listing but structures are only represented as images on the labels. Wikipedia has a useful unofficial collection with name-to-PubChem and DrugBank structure mappings but this is still being populated [40].

### Public versus commercial totals

As an adjunct to the individual comparisons we investigated overlaps for larger merges. By aggregating all the commercial sources in our 2006 study we obtained 1,711,674 with a collapse rate (compounds-in-common) of only 11%. Comparing this with 7,268,193 for PubChem gave an overlap of 524,083. The equivalent numbers for this 2008 study (there are some differences in sources but these are numerically small) are 2,284,464 for the commercial merge, also with an 11% collapse and 14,965,539 for PubChem. The two collections have 1,043,399 compounds in common. Thus 1,241,065 or approximately 65% of the compounds in these commercial collections are "outside" PubChem. The comparison between 2006 and 2008 not only shows increased overlap but also increased unique content in both sectors. This expanding complementarity is even more substantial considering the nesting of the 2.7 million Thompson Pharma commercial bioactive collection within PubChem that occurred between the two snapshots.

While a substantial proportion of compounds outside PubChem come from patents in GVKBIO they are nonetheless a rich source of bioactives. To put this in perspective an approximate maximum public bioactive count (i.e. compounds that would link to a publication and/or assay data within the system) was made by adding the following PubChem queries; KEGG, *Nature Chemical Biology*, *Drugs of the Future*, BindingDB, DrugBank, Protein 3D Structure, ChEBI, Pharmacological Action, PubMed via MeSH, PubMed and Active in any BioAssay. This produced 311,123 compounds, i.e. only 26% of the number outside PubChem and even these will contain a proportion of false positives from primary screens and molecular property assays. What should also not be overlooked for the exploration of bioactivity is the value of the negative data, particularly to discern structure-activity relationships, for those 637,022 compounds that have been tested but found to be inactive in the current assay collection.

## Conclusion

The expanded complementarity between public and commercial databases established in this work is a testimony to the vibrancy of the field. However, it does present users with the challenge of selecting sources whose utility best matches their technical and scientific objectives. There are, of course, many criteria that can be used for comparative evaluation. These include: coverage, data structure, searching options, export facilities, interface navigability, documentation, learning curve, update frequency, content quality, data mining features, connectivity with other sources as well as price and contractual restrictions for commercial products.

We suggest that such assessments inevitably remain incomplete without the direct comparison of compound content along the lines that we have reported. It should be pointed out that the determination of unique content and overlap both have high value. While the former can be conceived as an advantage it is important to understand the basis of this uniqueness before value can be ascribed. For example, it could mean that compounds have been extracted from some hitherto overlooked important source or, on the other hand, could have been generated as a consequence of heterogeneity and/or errors in structural representation. Two examples from this work highlight the issue. The GVKBIO data structure has the facility to select patent-only compounds. Consequently, our analysis was able to show that a substantial part of their unique content was derived from this source. While many of these have matches within PubChem, the entries do not currently include direct links to patents. In some cases such links are available indirectly via subscription databases. For DNP we were also able to show substantial unique content. While this natural product subset may have considerable value it is not clear which document selection or compound extraction strategies have given rise to this uniqueness.

A parsimonious interpretation of the overlap between databases might consider this as redundancy among public sources or "paying for the same stuff twice" in commercial ones. However, this overlooks one of the most important advances in contemporary informatics, namely the ability to synergistically combine data mining results between different sources that contain common entities, although this is often not a trivial exercise because of differences in data organisation and entity identifiers. In addition to largely standardised compound representations (SD files, SMILES or InChIs) some of the data sources described also contain common document (PubMed IDs or patent numbers) and protein identifiers (Swiss-Prot or Entrez Gene IDs) that can be extracted and cross-mapped.

There are two other advantages to determining common compound content. The first of these is extrinsic quality control, i.e. shared identical compounds are probably correct (the GVKBIO/WOMBAT concordance described above being a good example). The second is what could be termed orthogonal added value where complementary information can be combined for the same compounds even just by following out-links to different connecting databases. An example can be shown by querying PubChem for the structures in common between ChEBI, BindingDB and DrugBank. The resulting 133 compounds not only have a rich set of links from the three sources but also a fourth set within PubChem that includes BioAssay data for 119 of them.

In addition to the representation of chemical structure we suggest the curated links between compounds, proteins and documents constitute a very high intrinsic value for the databases that include them. Consequently, we have compared these where possible in this study. Whilst acknowledging the challenges of quantifying these relationships it is to be hoped that these kinds of metrics may become more widespread and standardised in the future.

While our results document the increase in public bioactives over the last two years, they also demonstrate the parallel expansion of commercial sources. Given that the latter are entirely derived from published documents or other non-confidential sources, the persistence of uniqueness shows the complementary scope of compound extractions and relationships captured by these products. However, the coverage pendulum will undergo a further swing in the public direction when the ChEMBL database comes on-line at the European Bioinformatics Institute towards the end of 2009 [41,42]. The result of a commercial-to-public transfer, this new source will include close to half a million compounds linked to protein identifiers and bioactivity information extracted from journal papers.

These developments currently present users with the best of both worlds. The academic sector and small companies who might not be able to afford commercial products have access to expanding resources, undreamt of even a few years ago, to enable their research in all areas of bioactive chemistry [9]. Even compound structures from patent documents, traditionally exclusively brokered by the commercial sector, have recently become accessible not only via the collaboration between Freepatentsonline [43], SureChem [37] and ChemSpider [4] but now also via ChEBI [14].

At the same time, companies or larger academic institutions that can invest in commercial databases to support drug discovery and/or chemical biology are likely to continue to do so, as long as vendors maintain content and functionality that are complementary to public sources. However, pharmaceutical companies in particular, are faced with the competitive necessity of exploiting both types of resource and integrating them efficiently. A good example of this in practice is the AstraZeneca implementation of the merged GVKBIO MedChem and Target Class products referred to in Methods. This currently includes out-links to PubMed for the scientific literature, MicroPatent [44] for patent documents, Entrez Gene for sequence identifiers, and PubChem CIDs as well as ChemSpider IDs for compound matches. Matches to AstraZeneca's Compound Collection are linked to internal screening data and some that do not match this collection but match compounds from preferred suppliers will have these links as well. This powerful combination not only takes full advantage of the overlap between the GVKBIO compounds and public sources but also the unique content quantified in this work.

## Competing interests

The authors declare that they have no competing interests.

## Authors' contributions

SM and CS developed the concept of the study. CS selected the public databases and subsets while SM selected the commercial databases and subsets. The data processing and result generation was performed by SM. CS drafted the manuscript and SM prepared the figures and tables. PV was responsible for developing the in-house merge of the GVKBIO databases and generating content statistics. All authors read and approved the final manuscript.

## Supplementary Material

Additional file 1 **The comparison matrix**. Table Three – The comparison matrix. Each heading corresponds to one of the data sets described in Methods. The 23 × 23 matrix can be reviewed from left to right across the columns and down the rows in database order. Click here for file
