# Supplementary material for: Quantitative assessment of the expanding complementarity between public and commercial databases of bioactive compounds
Source: J Cheminform. 2009 Jul 6;1:10. doi: 10.1186/1758-2946-1-10 (PMC3225862; doi:10.1186/1758-2946-1-10)
Supplement: Additional file 1 — The comparison matrix. Table Three – The comparison matrix. Each heading corresponds to one of the data sets described in Methods. The 23 × 23 matrix can be reviewed from left to right across the columns and down the rows in database order. [file 1758-2946-1-10-S1.doc]

## Table 3. The comparison matrix.

|  | GVKBIO | GVKBIO Journals | GVKBIO Patents | GVKBIO DD | GVKBIO CCD | Wombat | PubChem | PubChem Prous | PubChem PDB | PubChem actives | PubChem pharmacol | PubChem MLSMR | PubChem BindingDB | PubChem ChEBI | DrugBank | DrugBank approved | DrugBank experimental | DNP | MDDR | MDDR launched | BACE1 all | BACE1 journals | BACE1 patents |
| --- | --- | --- | --- | --- | --- | --- | --- | --- | --- | --- | --- | --- | --- | --- | --- | --- | --- | --- | --- | --- | --- | --- | --- |
| GVKBIO | 2054151 | 658198 | 1484218 | 2847 | 6178 | 171178 | 925845 | 3705 | 2864 | 1439 | 4332 | 6983 | 22388 | 3356 | 3165 | 1279 | 1723 | 19853 | 80756 | 1281 | 5228 | 389 | 4901 |
| GVKBIO Journals |  | 658198 | 88265 | 2779 | 5492 | 169734 | 361192 | 3510 | 2722 | 1358 | 4224 | 5672 | 21975 | 3257 | 3090 | 1263 | 1678 | 19175 | 33089 | 1257 | 552 | 389 | 225 |
| GVKBIO Patents |  |  | 1484218 | 1404 | 3149 | 45564 | 633115 | 1760 | 1132 | 879 | 2289 | 2908 | 8291 | 1257 | 1625 | 907 | 624 | 2330 | 60944 | 875 | 4934 | 92 | 4901 |
| GVKBIO DD |  |  |  | 3675 | 33 | 1060 | 3513 | 1191 | 290 | 468 | 1896 | 1147 | 218 | 722 | 1344 | 1170 | 177 | 504 | 1376 | 1230 | 2 | 1 | 1 |
| GVKBIO CCD |  |  |  |  | 8864 | 2652 | 7925 | 1446 | 235 | 200 | 753 | 258 | 421 | 118 | 327 | 26 | 137 | 710 | 6172 | 22 | 10 | 5 | 7 |
| Wombat |  |  |  |  |  | 180856 | 133124 | 1452 | 1263 | 752 | 1865 | 1943 | 14882 | 1155 | 1626 | 708 | 857 | 2376 | 15292 | 665 | 172 | 144 | 54 |
| PubChem |  |  |  |  |  |  | 14965539 | 4652 | 5706 | 7472 | 5311 | 233284 | 24203 | 7428 | 4370 | 1340 | 2826 | 81777 | 112515 | 1381 | 2007 | 170 | 1873 |
| PubChem Prous |  |  |  |  |  |  |  | 4652 | 252 | 316 | 1553 | 700 | 325 | 417 | 883 | 627 | 140 | 547 | 2339 | 916 | 26 | 23 | 6 |
| PubChem PDB |  |  |  |  |  |  |  |  | 5706 | 138 | 571 | 424 | 717 | 1007 | 1621 | 219 | 1426 | 889 | 655 | 161 | 12 | 9 | 4 |
| PubChem actives |  |  |  |  |  |  |  |  |  | 7472 | 716 | 5062 | 196 | 300 | 390 | 316 | 66 | 334 | 523 | 282 | 5 | 2 | 3 |
| PubChem pharmacol |  |  |  |  |  |  |  |  |  |  | 5311 | 1544 | 397 | 1318 | 1569 | 1168 | 356 | 1076 | 1747 | 1130 | 6 | 2 | 4 |
| PubChem MLSMR |  |  |  |  |  |  |  |  |  |  |  | 233284 | 445 | 811 | 965 | 753 | 202 | 1485 | 1101 | 662 | 6 | 3 | 3 |
| BindingDB |  |  |  |  |  |  |  |  |  |  |  |  | 24203 | 281 | 553 | 179 | 360 | 302 | 1777 | 150 | 121 | 86 | 62 |
| ChEBI |  |  |  |  |  |  |  |  |  |  |  |  |  | 7428 | 1302 | 543 | 779 | 2622 | 653 | 404 | 10 | 6 | 4 |
| DrugBank |  |  |  |  |  |  |  |  |  |  |  |  |  |  | 4545 | 1341 | 2999 | 941 | 1343 | 873 | 6 | 4 | 2 |
| DrugBank approved |  |  |  |  |  |  |  |  |  |  |  |  |  |  |  | 1341 | 61 | 225 | 884 | 837 | 1 | 1 | 0 |
| DrugBank experimental |  |  |  |  |  |  |  |  |  |  |  |  |  |  |  |  | 2999 | 725 | 326 | 55 | 5 | 3 | 2 |
| DNP |  |  |  |  |  |  |  |  |  |  |  |  |  |  |  |  |  | 144383 | 2771 | 166 | 21 | 15 | 6 |
| MDDR |  |  |  |  |  |  |  |  |  |  |  |  |  |  |  |  |  |  | 176600 | 1435 | 138 | 16 | 128 |
| MDDR launched |  |  |  |  |  |  |  |  |  |  |  |  |  |  |  |  |  |  |  | 1435 | 1 | 1 | 0 |
| BACE1 all |  |  |  |  |  |  |  |  |  |  |  |  |  |  |  |  |  |  |  |  | 5228 | 389 | 4901 |
| BACE1 journals |  |  |  |  |  |  |  |  |  |  |  |  |  |  |  |  |  |  |  |  |  | 389 | 62 |
| BACE1 patents |  |  |  |  |  |  |  |  |  |  |  |  |  |  |  |  |  |  |  |  |  |  | 4901 |

Each heading corresponds to one of the data sets described in Methods. The 23 x 23 matrix can be reviewed from left to right across the columns and down the rows in database order.
